# Working longer hours and body weight: An Australian study using household panel data (with measures of paid and unpaid time) to provide gender-specific estimates

**DOI:** 10.1016/j.ssmph.2023.101561

**Published:** 2023-11-20

**Authors:** L.S. Leach, T. Doan, L. Strazdins

**Affiliations:** National Centre for Epidemiology and Population Health (NCEPH), College of Health and Medicine, The Australian National University, Canberra, 2601, Australia

## Abstract

Time is a resource for health, and when time is constrained, people have less opportunity to maintain good health. This study focuses on the relationship between paid work hours (with a focus on long hours) and body weight for Australian men and women. Time is conceptualised as a 24-hour system, including time in paid work, time in unpaid work, and discretionary time (available for health promoting activities). We propose that to accurately estimate the relationship between long paid work hours and body weight, analyses need to take account of unpaid work hours, and that this is particularly important for women. Analyses utilised 16 waves of data from the Household, Income and Labour Dynamics in Australia (HILDA) panel study, with data on hours in paid and unpaid work and BMI at all waves (n = 113,084 observations, 54,664 from women and 58,424 from men). We used Mixed Effects models with a Two Stage Residual Inclusion (2SRI) approach to account for time unobserved heterogeneity and the reciprocity between time uses (paid and unpaid) and body weight. The results showed that for every 10 hours worked above the gender-specific average, women weighed 762 grms more and men weighed 1.34 kgs more. When the analyses were restricted to sedentary jobs this increased to 938 grms for women and 1.68 kgs for men. We contribute new evidence highlighting the importance of including unpaid work hours in research that aims to understand working time and health.

## Highlights

• Long hours in paid work is associated with higher body weight, for men and women
• Long hours in sedentary work is particularly detrimental to body weight
• Failing to include unpaid hours underestimates the impact of long paid work hours
• Policy and interventions need to tackle employment culture that promotes overwork

## 1 Introduction

### 1.1 Overview

Unhealthy body weight is a major public health issue in Australia and globally. Two in three Australian adults are overweight or obese (AIHW, 2023). OECD data from 2016 ranks Australia ninth worst out of 36 OECD countries for obesity (OECD, 2018). A standard approach to this problem is to promote behaviour change, such as improving nutrition and exercise (Sharma, 2007). This approach aims to influence energy consumption and expenditure via related behavioural mechanisms. While health behaviours are important proximal causes of weight gain leading to overweight and obesity, contextual social, economic and cultural factors are also critical in shaping the motivations and barriers that underpin why people do or don't (or can or can't) undertake positive health behaviours (Egger & Dixon, 2014).

In particular, the number of hours people spend at work influences the time available for health promoting activities (Artazcoz et al., 2009; Dixon et al., 2014; Doan et al., 2022). For example, Doan et al. show that as paid work hours increase, physical activity goes down (particularly for women) (Doan et al., 2022). Long hours on the job are incentivised by higher incomes and career progression (Artazcoz et al., 2009; Cha & Weeden, 2014; Dixon et al., 2014; Doan et al., 2022; Williams et al., 2008), creating a dynamic where healthy weight (which under the common health guidelines for Body Mass Index (BMI) is greater than 18.5 but less than 25) - or more specifically, the unallocated/discretionary time needed to maintain a healthy weight via exercise, sleep and preparing healthy meals – is potentially traded-off to spend more time at work. But how great is the relationship between overwork and body weight? Is the strength of this relationships similar for men and women? And what role might time spent in unpaid work play?

This paper investigates the consequences of overwork for body weight in Australia, and in doing so, recognises the role of time spent in unpaid domestic work. We outline that paid work is part of a 24-hour system that limits the discretionary time available for health promoting behaviour. Unpaid work hours are a critical component of this overall time use system, potentially amplifying the impact of paid work hours on body weight. The analyses utilised 16 waves of nationally representative Australian household panel data which uniquely include longitudinal measurements of time use both in- and outside of the labour market and adult BMI. Women and men were analysed separately to reflect gender differences in paid work hours, and time spent in unpaid work was factored into the estimates for weight. We investigate what happens to body weight (BMI) when men and women work long hours, as well as how sedentary work magnifies the impact.

### 1.2 Background

#### 1.2.1 The practice and lure of long work hours

The International Labour Standard *Hours of Work Industry Convention* states that work hours should be limited at 8 hours a day/48 hours per week (ILO – International Labour Organisation, 2018) although regulations differ between countries. Most countries define working more than 40 hours a week as overtime (ILO – International Labour Organisation, 2018) and occupational epidemiologists define 38–40 hours as a standard working week (i.e. Descatha et al., 2020; Li et al., 2020). But in practice, many people work longer than 40 hours a week (ILO – International Labour Organisation, 2018). In Australia, while a maximum of 38 hours is part of the National Employment Standards, many people work longer. OECD data indicates that 28% of women in Australia work 40+ hours in a usual working week, as do 56% of men (OECD, 2021). The lure to work long hours is strong, with a range of financial and professional incentives. Goldin argues that in many occupations the financial privilege that comes with extended hours on the job increases exponentially as hours lengthen, encouraging employees to work longer (Goldin, 2014). Aligning with this argument, Cha and Weeden (2014) show that the hourly pay for working fifty or more hours per week increased considerably in recent decades (relative to the pay of ordinary full-time work).

The lack of enforced regulation and the long hours often worked in Australia (Williams et al., 2008) runs counter to the research linking long hours to chronic health conditions such as coronary heart disease and poor mental health (et al., 2015; Virtanen et al., 2011). Research using joint estimates from the World Health Organisation and the International Labour Organisation found that in 2016, 8.9% of the world's population worked very long hours (55+), and that this contributed to 3.7% of deaths from ischemic heart disease and 6.0% from stroke (Pega et al., 2021).

#### 1.2.2 Evidence linking long work hours and body weight

The connection between long work hours and chronic health conditions likely stems, in part, from a link between long work hours and overweight/obesity. A 2013 systematic review found that 70% of studies reviewed reported a positive association between long work hours and obesity (Solovieva et al., 2013). A recent meta-analysis of 29 studies (n = 374,863) more confidently concluded that long work hours are associated with various adverse weight outcomes (including both overweight/obesity categories and other BMI change indicators) (Zhu et al., 2020). Several large longitudinal studies have similarly connected long work hours to increased weight gain. In pooled analysis of data from 19 different longitudinal cohort studies from Europe, US and Australia, working long hours was significantly associated with a small excess risk of overweight/obesity (Virtanen et al., 2020). Much of the existing research reports work hour risks in association with overweight and/or obese groups, with these categories largely based on Body Mass Index (BMI) measurements and standards. While the focus on overweight/obesity categories is warranted due to the increased health risks, there is also evidence that even small differences/increases in BMI (as a continuous measure) contribute to poorer health outcomes (i.e. Canoy et al., 2013). A limitation of much of the existing research is difficulty claiming causation due to health selection (Virtanen et al., 2020). People with overweight/obesity may not be able to work longer hours, have lower wages, and face employment discrimination (Hamermesh & Biddle, 1994; Morris, 2007). Methodologies and estimates that do not attempt to address reverse causation may misestimate the association between work hours and BMI.

There is evidence that jobs with large amounts of sedentary time amplify the effect of long work hours (Abramowitz, 2016; Lakdawalla & Philipson, 2007). Sedentary work not only involves low physical activity but facilitates greater consumption of high calorie, processed foods, a core reasons for rising obesity (Cutler et al., 2003). In research by Abramowitz (2016), longitudinal analyses of the American Time Use Survey found that every 10 additional hours of physically non-strenuous work was associated with a 0.42 increase in BMI (or 1.13 kgs) for women and 0.20 (or 0.64 kgs) for men.

While the literature is largely consistent in concluding that long work hours and sedentary work adversely impact weight, the magnitude of these effects and the extent to which they are stronger for women or men is unclear. Most ‘long hours’ study samples favour men, who are more likely to be working very long (paid) hours, and as such they may erroneously assume that work hours are largely a hazard for men (see Pega et al., 2021). The review by Solovieva et al. (2013) suggests that the relationship between long work hours and weight gain is greater for men, although the 2019 meta-analysis by Zhu et al. (2020) found similar positive associations for both women and men. Men and women have different labour market experiences, and women typically combine their paid hours with a much larger share of unpaid work (Baxter & Tai, 2016; Doan at el., 2021), a combination that may hide how paid work hours affects their body weight.

#### 1.2.3 The role of unpaid time and time constraints

Explicit investigation (or modelling) of the constraining effect of unpaid work on paid work, is largely absent from the existing research examining paid work hours and body weight. Although, some relevant research broadly supports the contribution of both work and family time as critical to body weight. For example, while Kramer and Chung's (2015) 16-year longitudinal study did not directly measure or model unpaid work hours, the authors used spouses' paid work hours as a proxy for time demands in the family domain. They found that BMI growth trajectory over time was hastened by not only an individual's work hours but also their spouse's work hours, as well as the number of children in the household. This finding implies that not only paid work, but also unpaid work, plays an important role in the development of higher BMI as they interact together to constrain overall time availability.

Other relevant work by Fan et al. (2015) investigates the hypothesis that gendered time-use pathways are important for body weight, by considering how both individuals' work hours and spouses' work hours impact physical activity and healthy eating. Although this study did not directly measure or model time spent in unpaid work, the authors found when women are part of a household with neo-traditional time use arrangements (where their male partner works longer paid hours than they do) they do less physical activity than women in couples with more equal time use. Whereas when men are a part of household with neo-traditional time use, they are able to do more physical activity. They conclude that the impacts of partner long work hours on women's physical activity is likely because women's time is more constrained due to family/caregiving responsibilities. This suggests the intersection between paid and unpaid work is important to consider, and model, in research examining the impacts of paid work on body weight.

A recent Australian study investigated physical activity as a function of both paid and unpaid time using a similar analytical approach as the current study to model the reciprocity/endogeneity between paid and unpaid hours. The results showed that as paid work increased, physical activity decreased (Doan et al., 2022). But more specifically, while higher paid and unpaid hours were associated with lower physical activity for women, both time uses had very minimal associations for men. The authors argue that the greater impact on women's physical activity is because their time is overall more committed, with less autonomy. While the relevant literature is limited, the above findings suggest it is important to consider unpaid time, and in particular the constraining effect of unpaid time on paid time, when estimating the relationship between paid work hours and body weight - particularly for identifying gender-specific associations.

### 1.3 Study aims

There is evidence that longer work hours lead to greater body weight. There are also indications that the intersection between paid and unpaid work should be accounted for in estimations of the size of this effect. Ignoring the reciprocity between paid and unpaid work may result in inaccurate estimates. This study used 16 waves of longitudinal representative Australian data to estimate differences in BMI when individuals work additional paid hours compared to the population average. While the focus is on long hours in paid work and the consequences for BMI, paid work was conceptualised as part of an overall time-use system. This time use system includes not only a) *paid work/time*, but also b) *unpaid/domestic work/time*, and c) *non-allocated/discretionary time (including time for health behaviours/personal care and sleep)* (Robinson, 1999). Mixed effect 2SRI models were used to accommodate the complex and reciprocal relationships between paid work hours, unpaid work hours, and BMI (addressing multiple sources of endogeneity) (Cerin & Leslie, 2008; Kim & von dem Knesebeck, 2018).

The primary hypothesis is that people who work long hours on the job, particularly in sedentary jobs, will have higher BMI's than those who work average hours (at the population level). It is additionally hypothesised that taking account of the reciprocal relationships between paid work, unpaid work and weight gain will show stronger associations than simple Random Effects (RE) model estimates that omit this reciprocity, particularly for women.

## 2 Methods

### 2.1 Data and analysis sample

The data analysed is from the Household, Income and Labour Dynamics in Australia (HILDA) survey. HILDA is an annual household-based survey of a nationally representative sample of Australians. The study commenced in 2001 and baseline data collection comprised of 13,969 participants from 7682 households. Participants (aged ≥15 years) within households are followed-up annually, with new people joining the study when they enter a participating household. Rates of retention are high (90–95% from wave to consecutive wave) (Wilkins et al., 2021). The survey combines detailed information on individual and household circumstances including measures of health, employment, income, time use and height and weight (used to calculate Body Mass Index (BMI)). For further detail see Watson and Wooden (2012)) and https://melbourneinstitute.unimelb.edu.au/hilda. The HILDA survey was approved by the Human Research Ethics Committee at the University of Melbourne.

BMI was not collected in the early waves of HILDA (waves 1–5), thus, the sample for the current study was restricted to waves 6–21 (2006–2021). To best capture the working population analyses were restricted to people aged 25–64 and individuals who were currently unemployed or not in the labour force were excluded from the analysis sample. About 1.2% of the sample with a BMI under 18.5 (underweight) were excluded (as the current study assumes that lower BMI scores represent healthier weight, and this is less likely to be the case for underweight individuals (e.g. Roh et al., 2014)). 10.4% of working people aged 25–64 who had missing BMI data were also excluded. The final analysis sample included 113,084 observations across 16 waves of data, 54,660 from women and 58,424 from men.

### 2.2 Measures

#### 2.2.1 Body Mass Index (BMI)

BMI is widely used as an indicator of body weight health/risk. The BMI measure was constructed from individual self-reported weight and height (i.e., weight (kg) divided by the square of height (metres)). Individuals provided height and weight information as part of the HILDA study self-completion questionnaire (see Wooden, 2008).

#### 2.2.2 Time use – work hours and unpaid time

*Paid workhours* were self-reported total hours per week usually worked in all jobs. Very high weekly workhours were top coded at 80h per week. Whilst the original measure of paid work hours in the HILDA survey is total hours worked per week, to reflect the current study's focus on *long work hours* the primary analyses report on an additional 10 h worked relative to the population average. For the gender stratified analyses, this population average is gender specific. See the Statistical Analyses section and Results section for further information.

#### 2.2.3 Unpaid workhours

Unpaid hours were self-reported as hours per week spent on household errands, housework, outdoor tasks, caring for children and disabled or elderly relatives, volunteering, and commuting time (as travel time is also committed time). This survey measure captures change longitudinally and is valid against Australian time diary data (Strazdins et al., 2016), although there is some evidence it overestimates childcare hours (Juster et al., 2003). We imputed ‘0’ unpaid work hours for employed people who did not report any domestic unpaid hours (8% of the sample). The total committed hours (paid and unpaid work) were top coded to a maximum of 126 h per week (or 18 per day, for 3% of the sample).

#### 2.2.4 Covariates

Models were adjusted for the following variables which may directly or indirectly affect BMI: *age* (and age squared); *sex* (men = 1); *marital status* (married or cohabitating couple = 1; others = 0); *ethnicity* (9 groups including Non-indigenous Australian; Aboriginal and Torres Strait Islander peoples; New Zealanders; Europeans; Middle East and North Africans; East and Southeast Asians; South and Central Asians; North and Latin Americans; Central and South Africans); *tertiary education* (yes/no); having *child(ren) under 6 years old* (yes/no); *living in owned house* (yes/no); *partnered and partner's working status* (no partner, a non-working partner, a working partner); having *long-term health conditions* (yes/no); *household socio-economic conditions* (equivalized household non-wage income, socio-economic disadvantage index (SEIFA – where higher scores represent higher SES)); *job characteristics* (work intensity - where higher scores represent higher job demands/intensity, work flexibility – where higher scores represent higher work flexibility, employment contract type, shift work schedule, occupation); *urban household* (yes/no), *state lived in* (8 states/territories) and *year* (13 years) dummies.

Supplementary analyses also stratified by sedentary vs. non-sedentary occupation type. Occupations were categorised into two broader groups: *Sedentary occupations* (including Managers, Professionals, and Clerical and Administrative Workers), and *non-sedentary occupations* (the remaining occupations).

### 2.3 Statistical approach

#### 2.3.1 Conceptualising a 24-hour system - time limit equations

Our model development starts with a time limit equation (1) to show how we conceptualise a constrained 24-hour time system including paid work time, unpaid domestic time and non-allocated time remaining for other (health promoting/personal care) activities. These groupings/domains are based on long-standing time use research (Robinson et al., 1999).

(1) That is: ***24 h = A + B***

Where ***A*** = paid hours + unpaid hours, and ***B*** = non-allocated time.

That is: ***24 h = [paid hours + unpaid hours] + [non-allocated time]***

We learn from the literature that BMI is affected by **B (**non-allocated time to undertake positive health behaviours).

(2) That is: ***BMI = f(B)***

These two equations (1), (2) bring us to a mediation model, where all committed hours (A-paid and unpaid) limit the time available to undertake **B** health promoting activities (in other words, B is affected by A) and this leads to BMI.

(3) That is: ***B = f(committed hours) = f(A)***

In this paper we are primarily interested in how paid work hours and unpaid domestic time affect BMI, so we can rewrite equation (1) as:

(4) ***B = 24h – A***

Then, we can combine equations (2), (4), to write equation (5) (to predict BMI):

BMI = f(B) = f(24h – A) = f(24h - paid hours - unpaid hours) = f(24h)-f(paid hours + unpaid hours)

(5) ***BMI***_***it***_ = ***α + β***_***1***_***.PaidHours***_***it***_***+ β***_***2***_***.UnpaidHours***_***it***_***+ λ.Xs***_***it***_***+ ε***_***it***_

Equation (5) is the reduced form equation for our modelling, where *PaidHours* and *UnpaidHours* are the variables of interest. We expect ***β***_***1***_ and ***β***_***2***_ to be positive as higher paid and unpaid time reduce unallocated time for health promoting activities (*B*) leading to higher BMI. Because 24 hours is a constant in the model (e.g., *f(24h) = α*) (and it is assumed that the health behaviour effects of non-allocated time operate via mediation) non-allocated time is not explicitly included in the estimation (i.e. equation (5)).

We add *Xs* as controlling variables including individual and household characteristics (e.g. *age, sex, ethnicity, education, marriage, long term health conditions, having young children, household socio-economic background*), job characteristics *(occupation, work intensity, work flexibility, work schedule, employment type),* and other variables capturing time and space effects *(urban, state and year dummies).* Adjustment for these covariates is based on prior Australian research showing they are important for work hours and/or BMI (e.g. Kifle & Desta, 2012; Taouk, Milner, & LaMontagne, 2019; Keramat et al., 2021). For example, Australia has a very high proportion of immigrants (about 30% born overseas) and also has a very large geographic coverage spanning over six states and two territories, with diverse labour market patterns in terms of wage income and workhours across location and ethnicity (Doan et al., 2023).

#### 2.3.2 Modelling approach

The modelling approach was tailored to the study aims - primarily to identify the impacts of long paid work hours on BMI, but also to show the importance of accounting for the time limiting effect of unpaid work. To do this, our modelling contrasts initial baseline random effect (RE) models with more comprehensive Mixed effect 2SRI models that: a) model the reciprocity between paid and unpaid work, b) control for observed factors in the data, c) account for other unobserved factors (i.e. heterogeneity that might influence the estimates), and d) reduce the influence of reverse causality between time uses and BMI.

Longitudinal Mixed effect models addressed the unobserved heterogeneity that affects both individual's time use and BMI (including both time invariant factors e.g. motivation, lifestyle, preferences, and time varying factors e.g. health shocks), however, because the Mixed effect model does not address reciprocal relationships (i.e. that work hours, unpaid hours and BMI may mutually affect each other), the Two Stage Residual Inclusion (2SRI)) approach was used in combination with the Mixed effect model in our current study. To be succinct, this is named in the results and tables ‘Mixed effect 2SRI model’.

#### 2.3.3 Mixed effect model with Two Stage Residual Inclusion (2SRI) approach

In the first stage of the 2SRI estimation, our identification strategy is to use exogenous factors that directly affect individual's time use, but do not *directly* impact BMI, to address the reciprocity in this relationship. More specifically, *paid work hours* were predicted using household non-wage income, as households with higher non-wage income are more likely to work fewer paid hours. *Unpaid time* was predicted using dummy variables representing *having children under 6 years old, house ownership status, and partner's employment status* since having young children may reflect a higher opportunity cost of labour market time; and young children, house ownership status and partner's employment status all affect individual's unpaid domestic work and then labour market time (Doan et al., 2022). Because the residuals/error terms in the BMI model are unlikely to be correlated (directly) with these factors (i.e. the orthogonal assumption), our model should be appropriately identified. Thus, following on from equation (5) above, in the first stage of the 2SRI we modelled:

(6) Paid hours = f(region's unemployment rate, household non-wage income, unpaid hours, controlling variables)

(7) Unpaid hours = f(having children under 6, house ownership, partner's status, paid hours, controlling variables)

Paid and unpaid time variables were entered in alternate equations to capture the interdependency in both forms of time use.

In the second stage of the 2SRI estimation, we modelled (equation (8)):

(8) BMI_it_ = α + β_1_.PaidHours_it_ + γ_1_(1^st^StageResiduals_of_PaidHours) + β_2_.UnpaidHours_it_ + γ_2_(1^st^StageResiduals_of_UnpaidHours) + λ.Xs_it_ + ε_it_

The 2SRI estimates are consistent, but as their standard errors are incorrect (Terza et al., 2008) the bootstrapping technique was applied (with 500 repetitions) to approximate the asymptotically standard errors.

The 2SRI estimator is similar to the 2SPS (two-stage predictor substitution for nonlinear models/generalized linear models, an extension of 2SLS estimator) except that in the second stage regression, the endogenous variables (paid and unpaid time) are not replaced by first-stage predictors (or predicted values of endogenous variables). Instead, first-stage residuals are included as additional regressors. We preferred the 2SRI model over other possible approaches (e.g. 2SLS or Instrumental variable estimator) as it can show the effect of unobserved confounders alongside the effect of workhours. The 2SRI enables us to model/include the unobserved confounders from the first stage, and thus the endogeneity of market and non-market is minimised. The 2SRI framework therefore explicitly accounts for endogeneity (i.e., the presence of unobservable confounders). The unobservable confounders could anbe reciprocal relationships (i.e. between paid and unpaid time) or omitted/unobserved factors that affect both time uses and BMI e.g., life style/individual attributes.

All of the variables used to predict time uses in the first stage of the mixed effect 2SRI model are strongly correlated with the time use variables (i.e., to satisfy the relevance assumption, see Supplementary Tables S1 and S2). They also theoretically meet the orthogonal/exclusion assumption - that is that they do not directly affect BMI, although this is untestable in 2SRI framework. For example, an individual's working status or preschool child does not *directly* affect their partner's body weight, even though these factors may *indirectly* affect body weight via the unpaid workload created by these factors. The exclusion or orthogonal assumption requires that the instruments are pre-determined or exogenous to the outcome (current BMI), For example, current BMI cannot operate backwards to influence the decision to have children in the past (although current BMI may affect current decisions to have children).

The analyses were initially conducted for the full sample, and following this were stratified by men and women. Further analyses were stratified by sedentary vs. non-sedentary work to investigate the extent to which long hours in inactive employment accelerates weight gain. To aid in interpreting the results, long weekly paid work hours are reported as an additional 10 hour increase relative to the population average. This average is 33 hours for women and 43 hours for men (as shown in Table 1), thus the gender-specific estimates for long work hours are when women work approximately 43 hours and men work 53 hours. By using gender-specific averages, the estimate is anchored at a point that is meaningful for men and women, as what constitutes ‘long work hours’ is context-based and different for men and women, particularly due to the time constraints of unpaid work. These thresholds align reasonably with the ILO working time limit of 8 hours a day/48 hours per week (ILO – International Labour Organisation, 2018).

**Table 1:** Descriptive statistics comparing working men and women, aged 25-64.

Variable	Weighted mean/%		Difference	P-value
	Women	Men		
BMI	27.00	27.58	−0.58	0.0000
Overweight (BMI ≥ 25) (yes = 1)	55.7%	70.2%	−0.15	0.0000
Obesity (BMI ≥ 30) (yes = 1)	25.8%	25.2%	0.01	0.0334
Have child(ren) under 6 (yes = 1)	18.0%	21.5%	−0.04	0.0000
Home ownership (yes = 1)	73.3%	70.7%	0.03	0.0000
*Partner status*				
Have no partner	28.6%	25.0%	0.04	0.0000
Have a non-working partner	6.4%	17.4%	−0.11	0.0000
Have a working partner	65.0%	57.6%	0.07	0.0000
Market/paid work hours	33.1	42.8	−9.70	0.0000
Unpaid domestic work hours	34.2	25.0	9.28	0.0000
Age	42.9	43.0	−0.07	0.9592
Marital status (married/de facto = 1)	73.7%	76.4%	−0.03	0.0000
Tertiary education (yes = 1)	41.4%	33.7%	0.08	0.0000
Long term health conditions (yes = 1)	17.5%	16.4%	0.01	0.0000
Equivalized non-wage income ($000)	14.9	15.1	−0.20	0.2216
Socio-economic index SEIFA (1–10)	6.02	5.94	0.08	0.0004
Urban (yes = 1)	86.9%	87.0%	0.00	0.3471
Work intensity (1–7)	4.8	4.73	0.07	0.0000
Work flexibility (1–7)	3.98	4.35	−0.37	0.0000
*Employment type*				
Fixed term	10.8	9.0	1.85	0.0000
Casual	16.7	11.1	5.65	0.0000
Permanent/ongoing	72.5	80.0	−7.50	0.0000
*Shift-work/work schedule*				
Regular day shift	79.4	78.9	0.53	0.0105
Regular night shift	3.7	3.7	0.02	0.0519
Irregular shifts	16.9	17.4	−0.55	0.0003

Note: HILDA 2006–2021 sample of 113,084 observations (54,664 women, 58,424 men). Estimates adjusted for sampling weights. Our models also controlled for ethnicity, occupation, state, and year dummies, but these are not reported in Table 1 to remain succinct. All monetary variables such as wage and income variables were discounted to the 2016 price level.

## 3 Results

### 3.1 Descriptive statistics

Descriptive statistics are presented in Table 1. In this sample of employed people aged 25–64, 55.7% of women and 70.2% of men met criteria for overweight (BMI ≥ 25), and about 25% of both men and women met criteria for obese (BMI ≥ 30). Women were more likely to have no partner or a working partner, while men were more likely to have a non-working partner. Men worked about 10 h more per week than women, and conversely women spent about 10 h more per week on domestic unpaid work (an average of 34 h for women and 25 h for men). Women were more likely to be tertiary educated than men, although they were also more likely to work in casual jobs (and less likely to have permanent employment).

### 3.2 Baseline (Random Effects - RE) estimates

The association between workhours and BMI was estimated using a RE model to provide baseline estimates controlling for observed characteristics of the individual, their household, job conditions, and location. The random effect estimates are presented in the first three columns of Table 2. Weekly work hours (WH) were not associated with BMI overall, and for both men and women. The association of unpaid time was also very small, despite statistical significance overall and for the men's model (at 5% and 10% level). Other findings to note include that socio-economic conditions, tertiary education, marital status, and long-term health condition were all associated with BMI.

**Table 2:** Estimates (overall, men and women) predicting BMI for RE model and Mixed Effect 2SRI model.

	RE estimates			Mixed effect 2SRI models – 2^nd^ stage		
	Overall	Men	Women	Overall	Men	Women
BMI rise by 10h increase in WH	0.010	0.011	0.006	0.312	0.423	0.281
Weight gain (kg) by 10h increase in WH	0.029	0.035	0.016	0.915	1.343	0.762
Key time variables						
Weekly workhours (WH)	0.001(0.0013)	0.0011(0.0017)	0.0006(0.0019)	0.0312**(0.0063)	0.0423**(0.0081)	0.0281**(0.0102)
First stage residual (WH)				−0.0313**(0.0065)	−0.0424**(0.0084)	−0.0284**(0.0104)
Weekly unpaid workhours (UPH)	−0.0015*(0.0007)	−0.0017+(0.001)	−0.0013(0.0009)	0.003(0.0026)	0.0018(0.0043)	0.0043(0.0035)
First stage residual (UPH)				−0.002(0.0027)	−0.0022(0.0044)	−0.0021(0.0035)
Covariates						
Age	0.259**(0.016)	0.251**(0.020)	0.276**(0.024)	0.235**(0.017)	0.212**(0.023)	0.259**(0.026)
Age squared	−0.002**(0.0002)	−0.002**(0.0002)	−0.003**(0.0003)	−0.002**(0.0002)	−0.002**(0.0003)	−0.002**(0.0003)
Sex (men = 1)	0.548**(0.087)			0.335**(0.102)		
Tertiary education (yes = 1)	−0.638**(0.080)	−0.507**(0.101)	−0.771**(0.118)	−0.670**(0.082)	−0.466**(0.102)	−0.850**(0.123)
Marital status (married/de facto = 1)	0.482**(0.048)	0.338**(0.069)	0.633**(0.066)	0.470**(0.051)	0.300**(0.072)	0.647**(0.072)
Long-term health condition (yes = 1)	0.181**(0.030)	0.102**(0.037)	0.248**(0.046)	0.212**(0.031)	0.141**(0.038)	0.280**(0.048)
SEIFA (1–10)	−0.039**(0.007)	−0.033**(0.010)	−0.046**(0.010)	−0.042*(0.007)	−0.034**(0.010)	−0.049**(0.011)
Work intensity (1–7)	−0.029**(0.009)	−0.029*(0.012)	−0.036**(0.013)	−0.067**(0.012)	−0.072**(0.015)	−0.075**(0.018)
Work flexibility (1–7)	0.006(0.008)	0.013(0.011)	0.003(0.012)	0.017*(0.008)	0.025*(0.011)	0.013(0.013)
Employment - Fixed term (base group)						
Casual	−0.081+(0.044)	−0.098+(0.058)	−0.052(0.063)	0.169*(0.069)	0.209*(0.085)	0.177(0.108)
Permanent or ongoing	0.022(0.032)	−0.032(0.041)	0.063(0.048)	0.018(0.033)	−0.024(0.041)	0.050(0.049)
Regular dayshift (base group)						
Regular night shift	−0.035(0.072)	−0.053(0.096)	−0.000(0.105)	0.025(0.072)	0.0002(0.097)	0.078(0.107)
Irregular shift	−0.055(0.036)	−0.112*(0.046)	0.006(0.054)	−0.060+(0.036)	−0.154**(0.047)	0.028(0.056)
Constant	20.33**(0.346)	21.37**(0.449)	19.70**(0.515)	19.67**(0.40)	20.35**(0.508)	18.98**(0.646)
Observations	90,593	44,587	46,006	87,543	43,665	43,878
Number of groups	14,648	7,118	7,530	14,344	7,035	7,309
Prob > Chi2	0.0000	0.0000	0.0000	0.0000	0.0000	0.0000

Note: Bootstrapped robust standard errors in parentheses (with 500 repetitions for 2SRI models), **p < 0.01, *p < 0.05, + p < 0.1 Standard errors can be converted to 95% Confidence Intervals: 95% CI Lower Bound = estimated coefficient–1.96*SE, 95% CI Upper Bound = estimated coefficient+1.96*SE. WH = paid workhours, UPH = unpaid workhours. All models controlled for occupation, ethnicity, urbanity, state, and year dummies. Predicted BMI mean: overall-27.5, Men-27.7, Women-27.2. Predicted body weight (kg) at mean BMI: Overall-80.6, Men-87.9, Women-73.8.

### 3.3 Mixed effect model with 2SRI framework

The results for the mixed effect models accounting for reciprocal relationships between paid work, unpaid work and BMI are shown in the last three columns of Table 2. Table 2 only displays the results for the second stage of the estimation (i.e., with BMI as the outcome). The complete results for both the first and second stages of the analyses can be seen in Supplementary Table S1 – where as expected, there was a strong inter-dependency between paid and unpaid work hours, such that higher weekly unpaid hours were associated with lower weekly paid work hours, with this relationship much greater for women (−0.139) than men (0.043).

The second stage findings in Table 2 confirm that on average over 16 years of data (2006–2021), long work hours were adversely associated with both men's and women's BMI. The estimates were strengthened compared to the prior RE model estimates which were small and insignificant, particularly for women. Overall, every 10 work hours more than the average was associated with 0.312 higher BMI points (0.915 kgs). When stratified by sex these results show 0.423 BMI points or 1.343 kgs higher for men, and 0.281 BMI points or 0.762 kgs higher for women (compared to gender-specific averages). Interestingly, the mixed effect 2SRI estimates show that the effect of unobserved confounders (coefficients of the first stage residual in Table 2) was negative and almost offset the positive (i.e., adverse) effect of workhours on BMI. In other words, if there were no unobserved confounders (including the reciprocity between paid and unpaid work), the RE would have also shown a strong effect of workhours on BMI. Because there are unobserved confounders, which net out the effect of workhours on BMI, conventional methods such as RE would appear to fail to correctly estimate the effect.

### 3.4 Sedentary vs non-sedentary long work hours and BMI

Further analyses explored differences in the association between work hours and BMI by sedentary and non-sedentary occupations (Supplementary Table S2). The Mixed effect 2SRI models were replicated for each of these sedentary occupation groups. Sedentary occupation workers’ BMI was more strongly associated with long workhours than non-sedentary occupation workers. An increase of 10 work hours more than the average hours was associated with 1.264 kgs higher for sedentary occupation workers, but only 0.416 kgs for non-sedentary occupation workers. For men in sedentary work, an increase of 10 work hours more than average was associated with 0.526 higher BMI points or a 1.68 kgs, whereas for men in non-sedentary work the magnitude is lower at 0.347 higher BMI points or 1.096 kg. Interestingly, for women in non-sedentary work there is virtually no effect of working longer hours, while there is a substantial effect for women in longer hours sedentary work (0.343 higher BMI points; 0.938 kgs). The complete results for both the first and second stages of the analyses can be seen in Supplementary Table S3.

## 4 Discussion

This study estimated the relationship between work hours and body weight using a holistic framework that positions time as a finite health resource. We considered and modelled the interdependence between paid work and family time to provide gender-aware estimates for both men and women. The findings show that working long hours on the job is associated with higher body weight, and that this is magnified when people work in occupations that are primarily sedentary. Although the baseline RE model failed to detect the effect of work hours on BMI, once reverse causality bias and unobserved heterogeneity were addressed, the association between workhours and body weight strengthened. While this was particularly the case for women, the estimate also strengthened for men. The additional risks related to sedentary work are shown in stratified analyses, where for workers in sedentary jobs, every 10 additional work hours was associated with an average gain of 1.68 kgs for men and 0.94 kgs for women.

The current findings confirm previous reviews (Solovieva et al., 2013), meta-analyses (Zhu et al., 2020) and longitudinal cohort studies (Courtemanche, 2009; Virtanen et al., 2020) demonstrating that working long hours leads to higher body weight. The findings also accord with prior research showing that this risk grows when people work long hours in sedentary jobs (Abramowitz, 2016; Lin et al., 2015). There is some uncertainty in the existing literature as to whether the amount of weight gained from overwork is relatively minor or substantial - as reflected by considerable heterogeneity between studies in meta-analyses conducted by Zhu et al. (2020). In the current study the estimates are significant at the population-level; particularly for sedentary work which adds 1.264 kgs for every additional 10 hours worked. The modelling approach explicitly captures the endogeneity and mutual influences on time (paid and unpaid) and BMI, increasing confidence in the estimates obtained.

Previous research has demonstrated that women's time, overall, is both more fragmented and constrained, with less autonomy than men's due to greater time spent in family/home-related work (Craig et al., 2012; Dominguez-Folgueras, 2022). In heterosexual couple-families women's time often extends further to provide a resource for their male partner (Doan et al., 2022; Fan et al., 2015). This is reflected in the first stage of the 2SRI models in the current study, where the estimates show that domestic/family-related variables (e.g., small children, having a working partner) impact women's unpaid time far more than men's, and that women are more likely to trade-off their paid work hours to spend time on unpaid tasks. The second stage of the 2SRI models shift to incorporate this unequal gendered exchange of paid and unpaid time to estimate the relationship between paid time and BMI. Once the capacity for unpaid work to reduce time in paid work is accounted for, we see a more robust and gender-balanced picture of the adverse impacts of overwork on body weight.

Given the gender-divide in long-hours paid work in Australia, and in all developed countries globally (Landivar, 2015; OECD, 2021), we might assume that the health trade-offs associated with working long hours are predominantly an issue for men's body weight. However, the current findings suggest this isn't the case. The results show that long hours in paid work is significantly associated with women's weight, and that this relationship is magnified when constraints on time outside of employment are accounted for. This is despite women's average paid work hours being lower than men's (in this sample 33 hours for women and 43 hours for men), providing further justification that what constitutes ‘long hours’ is contextual and is linked to other (unpaid) time commitments.

### 4.1 Study strengths and limitations

The current study included several important strengths. The data analysed were from a longitudinal, nationally representative, population-based sample of employed Australian adults, including robust measures of time use both in paid and unpaid work. Unpaid time is included in our conceptualization of how paid hours impact overall time availability, and this underpins the need for analyses that account for the gendered constraints of unpaid time. We use both theory and equations to describe a 24-hour time system and adopt a modelling approach that accounts for the reciprocity between paid and unpaid work and controls for both observed and unobserved influences.

While acknowledging the advance made by modelling unpaid hours in the home, there are some caveats on this measure. The measure of care and domestic time use was a self-reported survey measure, whereby hours were estimated for a usual week. Such survey measures are less precise than observational and diary assessments (Craig, 2007), but are robust for rankings within populations (Juster et al., 2003). Other work has validated the self-report unpaid time measure against diary estimates (Strazdins et al., 2016). Occupation was also self-reported in the HILDA study, and the broad stratification of occupations into sedentary and non-sedentary work is not sensitive to variation in sedentary/work activities within each occupation type.

In addition, BMI in the HILDA dataset was calculated using self-reported measures of height and body weight rather than measured objectively by an interviewer. While there is a very high correlation between self-reported and objective body measurements, it is also known that self-report measurements of weight are generally under-estimates (i.e., social desirability bias), especially as weight increases (Elgar & Stewart, 2008). If reported data is less accurate at the higher end of the body weight distribution, it may be that the results under-estimate the relationship between overwork and BMI. Wooden et al. (2008) show that HILDA Survey data on height and weight provides a distribution of BMI scores that compares reasonably well to the data collected by the ABS in the 2004-05 National Health Survey. Also, the current sample's overall pooled proportions for overweight (see Table 1: men 45% and women 30%) and obesity (men 25% and women 26%) are similar to those reported by the Australian Institute of Health and Welfare in 2014–2015 (overweight: men 42% and women 30%; obesity: men 28% and women 27%) (AIHW, 2017). We do however also note that BMI does not account for variations in body composition, is not able to differentiate between muscle and fat, and may misclassify some individuals. There are other measures which may better represent body fat content including waist-to-hip ratio, waist circumference and body fact percentage (Prentice & Jebb, 2001). While these were not available in the HILDA dataset across the period of investigation, there is a waist circumference measure in waves 13 and 17 which would support a future comprehensive investigation of potential measurement bias.

We did not attempt to directly model time spent in health-promoting activities (e.g., sleep, exercise, meal preparation), as detailed measures were not available. Instead, both theory and a holistic model of time-use were used to recognise that the relationship between paid work hours and BMI is a function of unpaid hours and discretionary/non allocated time. Further time-use research is needed explicitly measuring and modelling the influence and pathways via discretionary time allocation. For example, there is emerging evidence that incidental physical activity, or non-exercise activity thermogenesis (NEAT), is important for body weight (Levine et al., 2005). Measuring levels of incidental physical activity in unpaid work (including caregiving and household chores) vs. paid work could help explain health behaviour pathways to BMI. Furthermore, chronic health conditions are integral to both weight and work. While in this study, we control for the presence of existing long term health conditions, further research might consider how poor health might interact with paid and unpaid time use and BMI.

As the HILDA data is a longitudinal study, the sample in our study may have been impacted by non-response and attrition over time (for example at wave 19 only 60.7% of the original wave 1 sample remain). To reduce the impacts of declining representativeness in the sample, the study refreshes each year with any new members of the household aged≥15 joining the sample. Also, if a household member ‘splits off’ and leaves the original household, their new household joins the panel. In Wave 11 there was also a general top-up sample (of 4,096 individuals) to increase representation (particularly of new immigrants). Population sample weights are also available in the datasets to correct for non-response and attrition (Wilkins et al., 2021). Our own data investigations showed that the HILDA sample is fairly stable over 20 years, with little change in household socio-economic index (SEFIA), expected small increases in age, gradual small decreases in weekly work hours aligning broadly with labour market trends, and only small increases in BMI (aligning with increases in the proportion of Australians living with overweight and obesity reported by the AIHW– Australian Institute of Health and Welfare (2017; 2013). Our investigations did however show that those respondents missing BMI data tended to work slightly longer hours, were about 3.5 years younger and were from lower SEIFA households – a limitation that should be noted.

It is also important to note that while the analysis sample drawn from the HILDA dataset is broadly representative of employed Australian adults, the generalisability of our findings needs to be tested in both other Australian data as well as in other nations and cultural contexts. Results may differ in countries where the gender division in paid and unpaid hours is either more (e.g. Japan) or less polarised (e.g. Sweden) (OECD, 2021). We also acknowledge the gender-binary within the coding of the study sample. There were insufficient respondents with non-binary gender identifications to explore whether the links between paid and unpaid work hours and BMI might differ for this group.

Finally, we note that while the robust modelling in this paper is a strength, the 2SRI model does not offer tests for exclusion assumption to check the instruments used (in the first stage of the models) meet the orthogonal assumption. For this reason, we are cautious about claiming a causal association between paid work hours and greater BMI.

### 4.2 Conclusions

Health policy, research and clinical intervention largely assume that everyone has equal time to maintain and improve their health, but this is not the case - time itself is a critical resource and social determinant of health. The current findings indicate that time spent working long hours on the job comes at a health cost. Overwork is associated with greater body weight – with BMI levels significantly higher for both men and women when they work longer than average hours, particularly in sedentary work. Furthermore, we find evidence that the relationship between paid work hours and greater body weight is masked unless the additional time constraints of unpaid work are accounted for, particularly for women. Despite increases in medical and public health interventions targeting healthy body weight 39% of the global population remain overweight (WHO, 2021). Often people report that they know what they need to do to stay healthy – but that they can't find the time and opportunity to do so. It is critical to shift some of the burden of making time for health from individuals to broader employment and workplace policy and organisational reform, working together with policy organisations, industry, employers, and the public to reduce rates of overweight and obesity.

## Financial disclosure statement

The authors have no financial interests to disclose. The study was funded by an Australian Research Council Discovery Project Grant – DP190100975.

## Ethical statement

The content and analyses within this paper are the authors’ own original work. The paper uses until record data from the Household Income and Labour Dynamics in Australia (HILDA) Survey, 2006–2021. Data collection for the HILDA survey was approved by the Human Research Ethics Committee at the University of Melbourne.

The paper has not been previously published elsewhere and is not currently being considered for publication elsewhere.

## Author statement

Liana Leach: Conceptualization, Writing - Original draft preparation and Review and Editing, Methodology, Funding acquisition.

Tinh Doan: Conceptualization, Writing - Review and Editing, Methodology, Formal Analysis, Data Curation, Project administration.

Lyndall Strazdins: Conceptualization, Writing - Review and Editing, Methodology, Supervision, Funding acquisition.

## Declaration of interest

None.

## Data Availability

The authors do not have permission to share data.
