# Supplementary material for: Working longer hours and body weight: An Australian study using household panel data (with measures of paid and unpaid time) to provide gender-specific estimates
Source: SSM Popul Health. 2023 Nov 20;24:101561. doi: 10.1016/j.ssmph.2023.101561 (PMC10709171; doi:10.1016/j.ssmph.2023.101561)
Supplement: Multimedia component 1 [file mmc1.docx]

**Supplementary Tables**

**Table S1:** Predicting paid work hours (WH) and unpaid work hours (UPH) (1^st^ stage) and BMI (2^nd^ stage) with Mixed Effect 2SRI Modelling.

|  | Overall | | | Men | | | Women | | |
| --- | --- | --- | --- | --- | --- | --- | --- | --- | --- |
|  | (1) | (2) | (3) | (4) | (5) | (6) | (7) | (8) | (9) |
| Modelling stages | WH-1^st^ stage | UPH-1^st^ stage | 2nd stage | WH-1^st^ stage | UPH-1^st^ stage | 2nd stage | WH-1^st^ stage | UPH-1^st^ stage | 2nd stage |
| BMI rise by 10h increase in WH |  |  | 0.312 |  |  | 0.423 |  |  | 0.281 |
| Weight gain (kg) by 10h increase in WH |  |  | 0.915 |  |  | 1.343 |  |  | 0.762 |
| Key time variables |  |  |  |  |  |  |  |  |  |
| Weekly workhours (WH) |  | -0.1788** | 0.0312** |  | -0.1304** | 0.0423** |  | -0.2003** | 0.0281** |
|  |  | (0.0042) | (0.0063) |  | (0.0051) | (0.0081) |  | (0.0067) | (0.0102) |
| First stage residual (WH) |  |  | -0.0313** |  |  | -0.0424** |  |  | -0.0284** |
|  |  |  | (0.0065) |  |  | (0.0084) |  |  | (0.0104) |
| Weekly unpaid workhours (UPH) | -0.1062** |  | 0.0032 | -0.0426** |  | 0.0018 | -0.1386** |  | 0.0043 |
|  | (0.0027) |  | (0.0026) | (0.0037) |  | (0.0043) | (0.0034) |  | (0.0035) |
| First stage residual (UPH) |  |  | -0.0018 |  |  | -0.0022 |  |  | -0.0021 |
|  |  |  | (0.0027) |  |  | (0.0044) |  |  | (0.0035) |
| Instruments |  |  |  |  |  |  |  |  |  |
| Have a child under 6 (yes=1) |  | 11.6527** |  |  | 8.1149** |  |  | 14.9870** |  |
|  |  | (0.2223) |  |  | (0.2503) |  |  | (0.3553) |  |
| House ownership (living in own house=1) |  | 2.3342** |  |  | 2.1188** |  |  | 2.5232** |  |
|  |  | (0.1701) |  |  | (0.2081) |  |  | (0.2659) |  |
| [1] Have no partner (base group) |  |  |  |  |  |  |  |  |  |
| [2] Have a non-working partner |  | 1.2653* |  |  | 1.4379* |  |  | 2.2936** |  |
|  |  | (0.5387) |  |  | (0.6999) |  |  | (0.8262) |  |
| [3] Have a working partner |  | 2.5795** |  |  | 1.7467* |  |  | 3.5547** |  |
|  |  | (0.5228) |  |  | (0.6822) |  |  | (0.7920) |  |
| Household equivalized non-wage income ($’000) | -0.0045** |  |  | -0.0022+ |  |  | -0.0061** |  |  |
|  | (0.0010) |  |  | (0.0013) |  |  | (0.0013) |  |  |
| Covariates |  |  |  |  |  |  |  |  |  |
| Age | 0.5036** | 2.0956** | 0.2348** | 0.7739** | 1.7613** | 0.2115** | 0.2446** | 2.4479** | 0.2593** |
|  | (0.0441) | (0.0582) | (0.0174) | (0.0607) | (0.0721) | (0.0230) | (0.0621) | (0.0886) | (0.0261) |
| Age squared | -0.0064** | -0.0221** | -0.0021** | -0.0095** | -0.0185** | -0.0019** | -0.0035** | -0.0258** | -0.0023** |
|  | (0.0005) | (0.0007) | (0.0002) | (0.0007) | (0.0008) | (0.0003) | (0.0007) | (0.0010) | (0.0003) |
| Sex (men=1) | 7.7915** | -7.0171** | 0.3353** |  |  |  |  |  |  |
|  | (0.1520) | (0.1994) | (0.1020) |  |  |  |  |  |  |
| Tertiary education (yes=1) | 0.5802** | 0.8451** | -0.6700** | -0.4104 | 1.2954** | -0.4665** | 1.2422** | 0.6865* | -0.8497** |
|  | (0.1772) | (0.2165) | (0.0819) | (0.2512) | (0.2798) | (0.1025) | (0.2422) | (0.3161) | (0.1227) |
| Marital status | -0.5327** | 0.6279 | 0.4706** | 0.4645* | 0.7305 | 0.3006** | -1.4668** | 0.3491 | 0.6466** |
| (married/de facto=1) | (0.1272) | (0.5149) | (0.0510) | (0.1815) | (0.6758) | (0.0724) | (0.1731) | (0.7771) | (0.0724) |
| Long-term health condition | -0.7492** | -0.4667** | 0.2119** | -0.8747** | -0.1130 | 0.1413** | -0.6765** | -0.6234** | 0.2804** |
| (yes=1) | (0.0979) | (0.1432) | (0.0311) | (0.1380) | (0.1823) | (0.0385) | (0.1374) | (0.2173) | (0.0483) |
| SEIFA (1-10) | 0.0440* | 0.0104 | -0.0419** | 0.0255 | -0.0188 | -0.0342** | 0.0656* | 0.0314 | -0.0496** |
|  | (0.0214) | (0.0308) | (0.0076) | (0.0295) | (0.0376) | (0.0101) | (0.0299) | (0.0478) | (0.0112) |
| Work intensity (1-7) | 1.1245** |  | -0.0672** | 1.0019** |  | -0.0722** | 1.2043** |  | -0.0747** |
|  | (0.0335) |  | (0.0117) | (0.0480) |  | (0.0148) | (0.0457) |  | (0.0184) |
| Work flexibility (1-7) | -0.3566** |  | 0.0170* | -0.2670** |  | 0.0247* | -0.3869** |  | 0.0134 |
|  | (0.0316) |  | (0.0086) | (0.0448) |  | (0.0111) | (0.0431) |  | (0.0127) |
| [1] Employment - Fixed term (base group) |  |  |  |  |  |  |  |  |  |
| [2] Casual | -8.1526** |  | 0.1691* | -7.2151** |  | 0.2088* | -8.4474** |  | 0.1771 |
|  | (0.2096) |  | (0.0691) | (0.3507) |  | (0.0853) | (0.2569) |  | (0.1081) |
| [3] Permanent or ongoing | 0.0341 |  | 0.0186 | -0.0324 |  | -0.0235 | 0.1158 |  | 0.0500 |
|  | (0.1290) |  | (0.0329) | (0.1899) |  | (0.0413) | (0.1726) |  | (0.0497) |
| [1] Regular dayshift (base group) |  |  |  |  |  |  |  |  |  |
| [2] Regular night shift | -2.4103** |  | 0.0249 | -1.8163** |  | 0.0002 | -2.9046** |  | 0.0782 |
|  | (0.2697) |  | (0.0721) | (0.4017) |  | (0.0968) | (0.3497) |  | (0.1072) |
| [3] Irregular | 0.0263 |  | -0.0599+ | 0.7728** |  | -0.1539** | -0.7206** |  | 0.0277 |
|  | (0.1547) |  | (0.0364) | (0.2337) |  | (0.0466) | (0.2040) |  | (0.0560) |
| Constant | 28.5431** | -13.1844** | 19.6676** | 28.9168** | -15.2215** | 20.3484** | 35.4910** | -20.4923** | 18.9860** |
|  | (0.9623) | (1.2569) | (0.3977) | (1.3026) | (1.5316) | (0.5081) | (1.3637) | (1.9343) | (0.6464) |
| Observations | 92,525 | 147,983 | 87,543 | 45,263 | 72,897 | 43,665 | 47,262 | 75,086 | 43,878 |
| Number of unique individuals | 14,793 | 19,391 | 14,344 | 7,171 | 9,507 | 7,035 | 7,622 | 9,884 | 7,309 |

*Note: Bootstrapped robust SEs in parentheses with 500 repetitions, ** p<0.01, * p<0.05, + p<0.1. WH= paid workhours, UPH=unpaid workhours. All models controlled for occupation, ethnicity, urbanity, state, and year dummies.*

**Table S2:** Mixed effect model with 2SRI estimates by occupations (sedentary vs non-sedentary occupation) and gender (2^nd^ stage)*.*

|  | Sedentary Occupations | | | Non-sedentary Occupations | | |
| --- | --- | --- | --- | --- | --- | --- |
|  | Overall | Men | Women | Overall | Men | Women |
| BMI gain by 10h increase in WH | 0.431 | 0.526 | 0.343 | 0.142 | 0.347 | -0.010 |
| Weight gain by 10h increase in WH (kg) | 1.264 | 1.680 | 0.938 | 0.416 | 1.096 | -0.027 |
| Key time variables |  |  |  |  |  |  |
| Weekly workhours | 0.0431** (0.008) | 0.0526**(0.011) | 0.0343**(0.012) | 0.0142+(0.008) | 0.0347**(0.010) | -0.001(0.015) |
| First stage residual (WH) | 0.039**(0.008) | -0.046**(0.012) | 0.031**(0.018) | -0.019*(0.009) | -0.039**(0.011) | -0.005(0.015) |
| Weekly unpaid workhours | 0.005+(0.0028) | 0.003(0.005) | 0.004(0.003) | 0.006(0.005) | 0.006(0.006) | 0.008(0.007) |
| First stage residual (UPH) | -0.002(0.0028) | -0.005(0.005) | -0.001(0.003) | -0.006(0.005) | -0.005(0.006) | -0.009(0.007) |
| Covariates |  |  |  |  |  |  |
| Age | 0.226**(0.023) | 0.180**(0.031) | 0.266**(0.031) | 0.252**(0.0273) | 0.247**(0.032) | 0.260**(0.049) |
| Age2 | -0.002**(0.0003) | -0.001**(0.0003) | -0.002**(0.0004) | -0.002**(0.0003) | -0.002**(0.0004) | -0.003**(0.001) |
| Sex (men=1) | 0.492**(0.118) |  |  | 0.203(0.153) |  |  |
| Tertiary education (yes=1) | -0.695**(0.097) | -0.619**(0.124) | -0.744**(0.142) | -0.611**(0.131) | -0.345*(0.172) | -0.842**(0.198) |
| Marital status (married/de facto=1) | 0.508**(0.062) | 0.365**(0.100) | 0.626**(0.080) | 0.438**(0.080) | 0.272**(0.099) | 0.666**(0.131) |
| Long-term health condition (yes=1) | 0.184**(0.041) | 0.072(0.053) | 0.249**(0.058) | 0.263**(0.049) | 0.193**(0.056) | 0.393**(0.088) |
| SEIFA (1-10) | -0.040**(0.009) | -0.037**(0.013) | -0.042**(0.012) | -0.041**(0.013) | -0.024(0.015) | -0.067**(0.024) |
| Work intensity (1-7) | -0.076**(0.017) | -0.079**(0.023) | -0.076**(0.023) | -0.062**(0.016) | -0.079**(0.019) | -0.056*(0.027) |
| Work flexibility (1-7) | 0.007 (0.011) | 0.018 (0.016) | 0.001 (0.015) | 0.023+ (0.013) | 0.037*(0.016) | 0.010 (0.023) |
| Employment - Fixed term (base group) |  |  |  |  |  |  |
| Casual | 0.251**(0.097) | 0.218(0.144) | 0.223+(0.130) | 0.016(0.099) | 0.162(0.112) | -0.072(0.175) |
| Permanent or ongoing | 0.005(0.038) | -0.052(0.049) | 0.041(0.055) | 0.038(0.064) | 0.024(0.072) | 0.079(0.116) |
| Regular dayshift (base group) |  |  |  |  |  |  |
| Regular night shift | -0.066(0.135) | -0.097(0.193) | -0.052(0.183) | 0.017(0.084) | 0.003(0.107) | 0.055(0.133) |
| Irregular shift | 0.053(0.054) | -0.056(0.064) | 0.110(0.081) | -0.087+(0.052) | -0.169**(0.065) | -0.005(0.085) |
| Constant | 19.103** | 20.593** | 18.384** | 20.343** | 19.884** | 20.398** |
|  | (0.507) | (0.674) | (0.760) | (0.615) | (0.714) | (1.165) |
| Observations | 51,261 | 21,751 | 29,510 | 36,282 | 21,914 | 14,368 |
| Number of unique individuals | 9,352 | 3,994 | 5,358 | 8,205 | 4,662 | 3,543 |

Note: *Bootstrapped robust standard errors in parentheses with 500 repetitions, ** p<0.01, * p<0.05, + p<0.1. Standard errors can be converted to 95% Confidence Intervals: 95% CI Lower Bound = estimated coefficient–1.96*SE, 95% CI Upper Bound = estimated coefficient+1.96*SE. WH=paid workhours, UPH=unpaid workhours. All models controlled further for ethnicity, occupation within each broader occupation group, urbanity, state, and year dummies*

**Table S3**: Mixed effect 2SRI models (1^st^ and 2^nd^ stage) by occupation type (sedentary vs non-sedentary occupation) and gender*.*

|  | Sedentary occupations | | | | | | Non-sedentary occupations | | | | | |
| --- | --- | --- | --- | --- | --- | --- | --- | --- | --- | --- | --- | --- |
|  | Men | | | Women | | | Men | | | Women | | |
|  | WH-1^st^ stage | UPH-1^st^ stage | 2nd stage | WH-1^st^ stage | UPH-1^st^ stage | 2nd stage | WH-1^st^ stage | UPH-1^st^ stage | 2nd stage | WH-1^st^ stage | UPH-1^st^ stage | 2nd stage |
| BMI gain by 10h increase in WH |  |  | 0.526 |  |  | 0.343 |  |  | 0.347 |  |  | -0.010 |
| Kg gain by 10h increase in WH |  |  | 1.680 |  |  | 0.938 |  |  | 1.096 |  |  | -0.027 |
| Key time variables |  |  |  |  |  |  |  |  |  |  |  |  |
| Weekly workhours |  | -0.1405** | 0.0526** |  | -0.3477** | 0.0343** |  | -0.1232** | 0.0347** |  | -0.1667** | -0.0010 |
|  |  | (0.0108) | (0.0112) |  | (0.0119) | (0.0115) |  | (0.0062) | (0.0101) |  | (0.0098) | (0.0145) |
| First stage residual (WH) |  |  | -0.0464** |  |  | -0.0311** |  |  | -0.0392** |  |  | -0.0051 |
|  |  |  | (0.0116) |  |  | (0.0117) |  |  | (0.0105) |  |  | (0.0150) |
| Weekly unpaid workhours | -0.0438** |  | 0.0028 | -0.1523** |  | 0.0044 | -0.0411** |  | 0.0056 | -0.1033** |  | 0.0075 |
|  | (0.0051) |  | (0.0052) | (0.0042) |  | (0.0034) | (0.0050) |  | (0.0062) | (0.0050) |  | (0.0074) |
| First stage residual (UPH) |  |  | -0.0046 |  |  | -0.0006 |  |  | -0.0046 |  |  | -0.0086 |
|  |  |  | (0.0053) |  |  | (0.0034) |  |  | (0.0063) |  |  | (0.0074) |
| Instruments |  |  |  |  |  |  |  |  |  |  |  |  |
| Have a child under 6 (yes=1) |  | 8.7728** |  |  | 18.7979** |  |  | 7.7636** |  |  | 10.5066** |  |
|  |  | (0.3390) |  |  | (0.4537) |  |  | (0.3513) |  |  | (0.4833) |  |
| House ownership |  | 2.1068** |  |  | 2.3502** |  |  | 2.0005** |  |  | 2.0665** |  |
| (living in own house=1) |  | (0.2797) |  |  | (0.3338) |  |  | (0.2808) |  |  | (0.3699) |  |
| [1] *Have no partner (base group)* |  |  |  |  |  |  |  |  |  |  |  |  |
| [2] Have a non-working partner |  | 1.9072+ |  |  | 3.2594** |  |  | 1.2911 |  |  | 1.8330 |  |
|  |  | (1.1093) |  |  | (1.1018) |  |  | (0.9041) |  |  | (1.1558) |  |
| [3] Have a working partner |  | 2.0789+ |  |  | 4.0253** |  |  | 1.7075+ |  |  | 3.3705** |  |
|  |  | (1.0609) |  |  | (1.0114) |  |  | (0.8957) |  |  | (1.1326) |  |
| Household equivalized | -0.0003 |  |  | -0.0045** |  |  | -0.0072* |  |  | -0.0138** |  |  |
| non-wage income ($’000) | (0.0012) |  |  | (0.0015) |  |  | (0.0031) |  |  | (0.0027) |  |  |
| Covariates |  |  |  |  |  |  |  |  |  |  |  |  |
| Age | 0.7898** | 2.0656** | 0.1799** | 0.1448+ | 3.0242** | 0.2660** | 0.6765** | 1.6033** | 0.2471** | 0.3779** | 1.6761** | 0.2601** |
|  | (0.0808) | (0.1068) | (0.0311) | (0.0760) | (0.1207) | (0.0313) | (0.0837) | (0.0908) | (0.0324) | (0.0993) | (0.1198) | (0.0488) |
| Age squared | -0.0094** | -0.0218** | -0.0014** | -0.0024** | -0.0327** | -0.0023** | -0.0086** | -0.0167** | -0.0023** | -0.0051** | -0.0175** | -0.0025** |
|  | (0.0010) | (0.0012) | (0.0003) | (0.0009) | (0.0014) | (0.0004) | (0.0010) | (0.0010) | (0.0004) | (0.0012) | (0.0013) | (0.0006) |
| Tertiary education (yes=1) | -0.2850 | 1.1856** | -0.6194** | 0.6390* | 0.6198 | -0.7437** | -2.1143** | 1.7327** | -0.3452* | 0.3059 | -0.4957 | -0.8421** |
|  | (0.2681) | (0.3536) | (0.1241) | (0.2679) | (0.3784) | (0.1417) | (0.4362) | (0.4348) | (0.1724) | (0.4229) | (0.4788) | (0.1982) |
| Marital status | 0.0355 | 0.0198 | 0.3654** | -1.5842** | -0.5319 | 0.6263** | 0.9394** | 1.1996 | 0.2724** | -1.3813** | 0.7324 | 0.6663** |
| (married/de facto=1) | (0.2363) | (1.0683) | (0.1000) | (0.1987) | (0.9808) | (0.0798) | (0.2464) | (0.8866) | (0.0994) | (0.2952) | (1.1187) | (0.1308) |
| Long-term health condition | -0.5895** | 0.4743+ | 0.0721 | -0.6919** | -0.3097 | 0.2487** | -1.1850** | -0.3668 | 0.1928** | -0.6247** | -0.6326* | 0.3927** |
| (yes=1) | (0.1818) | (0.2622) | (0.0527) | (0.1718) | (0.2875) | (0.0578) | (0.1966) | (0.2365) | (0.0564) | (0.2226) | (0.3015) | (0.0884) |
| SEIFA (1-10) | 0.0877* | -0.0693 | -0.0372** | 0.0390 | -0.0125 | -0.0418** | -0.0369 | 0.0033 | -0.0237 | 0.0933+ | 0.0020 | -0.0666** |
|  | (0.0390) | (0.0520) | (0.0133) | (0.0335) | (0.0617) | (0.0121) | (0.0419) | (0.0496) | (0.0151) | (0.0543) | (0.0653) | (0.0239) |
| Work intensity (1-7) | 1.3505** |  | -0.0790** | 1.3755** |  | -0.0757** | 0.7125** |  | -0.0786** | 0.7891** |  | -0.0563* |
|  | (0.0666) |  | (0.0232) | (0.0566) |  | (0.0231) | (0.0641) |  | (0.0189) | (0.0707) |  | (0.0271) |
| Work flexibility (1-7) | -0.2837** |  | 0.0184 | -0.3912** |  | 0.0012 | -0.2691** |  | 0.0373* | -0.2931** |  | 0.0095 |
|  | (0.0621) |  | (0.0158) | (0.0531) |  | (0.0154) | (0.0605) |  | (0.0156) | (0.0677) |  | (0.0226) |
| [1] Employment - Fixed term (base group) |  |  |  |  |  |  |  |  |  |  |  |  |
| [3] Casual | -9.2236** |  | 0.2178 | -9.0539** |  | 0.2232+ | -7.0028** |  | 0.1619 | -7.7331** |  | -0.0715 |
|  | (0.7788) |  | (0.1440) | (0.3463) |  | (0.1297) | (0.4177) |  | (0.1117) | (0.4002) |  | (0.1749) |
| [3] Permanent or ongoing | 0.5535* |  | -0.0521 | 0.2639 |  | 0.0405 | -0.7344* |  | 0.0243 | -0.1091 |  | 0.0786 |
|  | (0.2373) |  | (0.0487) | (0.2021) |  | (0.0554) | (0.2929) |  | (0.0722) | (0.3088) |  | (0.1155) |
| [1] Regular dayshift (base group) |  |  |  |  |  |  |  |  |  |  |  |  |
| [2] Regular night shift | -2.9207** |  | -0.0968 | -3.8035** |  | -0.0518 | -1.2943** |  | 0.0032 | -1.8716** |  | 0.0545 |
|  | (0.8371) |  | (0.1934) | (0.5990) |  | (0.1830) | (0.4498) |  | (0.1066) | (0.4224) |  | (0.1334) |
| [2] Irregular shift | 0.2778 |  | -0.0558 | -2.0209** |  | 0.1095 | 1.2478** |  | -0.1697** | 0.7862** |  | -0.0046 |
|  | (0.3698) |  | (0.0644) | (0.2919) |  | (0.0808) | (0.2973) |  | (0.0645) | (0.2690) |  | (0.0850) |
| Constant | 26.785** | -21.919** | 20.593** | 38.012** | -25.993** | 18.384** | 31.283** | -11.523** | 19.884** | 29.697** | -3.109 | 20.398** |
|  | (1.7423) | (2.2727) | (0.6739) | (1.6231) | (2.5680) | (0.7600) | (1.7706) | (1.9529) | (0.7141) | (2.2738) | (2.6874) | (1.1650) |
| Observations | 22,449 | 30,708 | 21,751 | 31,605 | 37,908 | 29,510 | 22,814 | 42,189 | 21,914 | 15,657 | 37,178 | 14,368 |
| Number of groups | 4,070 | 5,053 | 3,994 | 5,579 | 6,217 | 5,358 | 4,764 | 7,339 | 4,662 | 3,743 | 7,092 | 3,543 |

Note: *Bootstrapped robust standard errors in parentheses with 500 repetitions, ** p<0.01, * p<0.05, + p<0.1. WH=paid workhours, UPH=unpaid workhours. All models controlled further for ethnicity, occupation within each broader occupation group, urbanity, state and year dummies.*
